# Allicin ameliorates acute myocardial infarction in rats by modulating calcium homeostasis in cardiomyocytes through the induction of hydrogen sulfide production

**DOI:** 10.3389/fphar.2025.1557685

**Published:** 2025-03-26

**Authors:** Weiyu Liu, Shaojun Xu, Juan Wang, Xinxia Li, Ruiting Liu, Le Zhao, Yikui Li, Rongmei Shi, Jinyan Zhang

**Affiliations:** ^1^ Beijing Key Laboratory of Pharmacology of Chinese Materia Medic, Institute of Basic Medical Sciences of Xiyuan Hospital, China Academy of Chinese Medical Sciences, Beijing, China; ^2^ College of Pharmacy, Xinjiang Medical University, Urumqi, China; ^3^ Xinjiang Key Laboratory of Garlic Medicinal Research, Urumqi, China; ^4^ Health Prevention Department, Xiyuan Hospital, China Academy of Chinese Medical Sciences, Beijing, China

**Keywords:** allicin, myocardial infarction, hydrogen sulfide, calcium homeostasis, cardiomyocytes

## Abstract

**Background:**

Acute myocardial infarction (AMI) is a common cardiovascular disease with high morbidity and mortality rates. Allicin, the primary active component of traditional Chinese herbs garlic, has multiple cardiovascular effects. However, the protective effect of allicin on AMI is rare. This study aimed to identify the pathways through which allicin stimulates hydrogen sulfide (H_2_S) production to regulate calcium ion (Ca^2+^) homeostasis in cardiomyocytes, thereby contributing to AMI protection.

**Methods:**

In this study, we established an AMI rat model by ligating the left anterior descending branch of the coronary artery to assess the therapeutic effect of allicin. We also investigated its influence on cardiomyocyte Ca^2+^ homeostasis. To determine the role of H_2_S production in the effects of allicin, we identified the H_2_S synthase in healthy rat myocardial tissue and serum and then applied H_2_S synthase inhibitors to block H_2_S production.

**Results:**

The results indicate that allicin significantly enhanced cardiac function, raised H_2_S levels in myocardial tissue and serum, reduced necrosis tissue size, decreased myocardial enzyme levels, and improved myocardial pathological changes. Surprisingly, allicin also notably increased H_2_S synthase levels. These findings suggest that allicin shields AMI rats by stimulating H_2_S production, acting both as a direct H_2_S donor and indirectly boosting H_2_S synthase expression. Furthermore, allicin enhanced Ca^2+^ homeostasis in cardiomyocytes by improving cardiomyocyte contraction kinetics and regulating the function and expression of key proteins related to Ca^2+^ transport in cardiomyocytes. The effect of allicin on Ca^2+^ homeostasis was partially decreased but not entirely abolished when H_2_S production was inhibited using H_2_S synthase inhibitors PAG and AOAA. This suggests that while the impact of allicin is strongly associated with H_2_S, additional independent mechanisms are also involved.

**Conclusion:**

Our study presents novel evidence demonstrating that allicin modulates Ca^2+^ homeostasis in cardiomyocytes by stimulating H_2_S production, thereby conferring protection against AMI. Furthermore, the protective effects of allicin are partly mediated by, but not solely reliant on, the generation of H_2_S. These findings not only provide mechanistic insights into the anti-AMI effects of allicin but also underscore its therapeutic promise.

## Graphical Abstract

**Figure:**
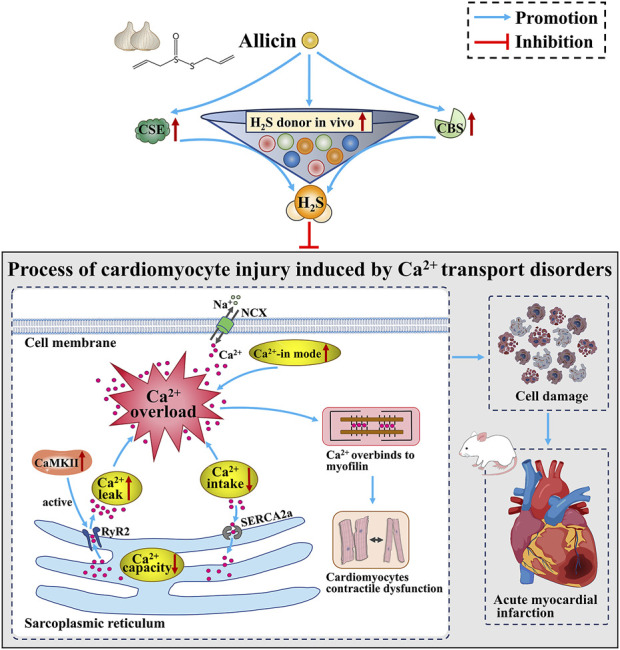
Allicin ameliorates acute myocardial infarction in rats by modulating calcium homeostasis in cardiomyocytes through the induction of hydrogen sulfide production.

## 1 Introduction

Acute myocardial infarction (AMI) poses a significant global health concern due to its high morbidity rates, rapid disease progression, and increased mortality (Alexander et al., 2017; McAloon et al., 2016). While percutaneous coronary intervention (PCI) and the integration of Western medicine have improved the survival rates of AMI patients, barriers persist in the revascularization process, resulting in irreversible damage to myocardial tissues and cells, thereby affecting patient prognosis (Braunwald and Kloner, 1985; Yellon and Hausenloy, 2007). Pathologically, AMI involves the damage of many cardiac cells following a sudden ischemic insult (Chang et al., 2019). Recent research indicates that alterations in the concentration of calcium ions (Ca^2+^) within cardiomyocytes are the primary cause of cardiomyocyte damage post-AMI. Ca^2+^ serves as an omnipresent intracellular messenger, regulating numerous cellular functions, with Ca^2+^ overload potentially leading to cell death (Bagur and Hajnóczky, 2017).

The regulation of Ca^2+^ homeostasis in cardiomyocytes is a complex and extensive system. Ca^2+^ flows in and out of the cell and is transported between different organelles and between organelles and the cytoplasm. This process involves the transfer of Ca^2+^ into the cell *via* ion channels on the cytosolic membrane, the removal of Ca^2+^ from the cell by ion pumps on the cytosolic membrane, the release of Ca^2+^ from the sarcoplasmic reticulum (SR) Ca^2+^ pool, and the reuptake and storage of Ca^2+^ by the SR Ca^2+^ pool, among others. Moreover, each of these processes is strictly regulated by numerous Ca^2+^ channels, Ca^2+^ pumps, and key Ca^2+^-transporting proteins. Any abnormality in these processes can alter the concentration of Ca^2+^ in the cytoplasm, leading to abnormalities in the diastolic and contractile amplitude and rhythm of cardiomyocytes, ultimately resulting in cardiomyocyte damage and apoptosis.

In AMI, a small quantity of Ca^2+^ is admitted into the cardiomyocytes *via* L-type Ca^2+^ channels, initiating substantial Ca^2+^ release from the SR, resulting in Ca^2+^ overload. This, in turn, compromises the contractile function of cardiomyocytes and could potentially induce cell apoptosis (Zhang et al., 2018). Thus, the transportation of Ca^2+^ and intracellular Ca^2+^ homeostasis represent attractive and promising targets for pharmacological interventions aiming at cardiac protection. With the advancement of traditional Chinese medicine, attention has been given to the prevention and treatment of AMI using these methods (Han et al., 2019). Allicin (C_6_H_10_S_2_O), the main pharmacologically active compound found in garlic (*Allium sativum* L.), a traditional Chinese medicine (Lawson and Hunsaker, 2018), has exhibited various cardiovascular protective effects, such as blood pressure reduction (Cui et al., 2020), blood lipid regulation (Chan et al., 2013), and prevention of atherosclerosis (Lei et al., 2010). However, only a limited number of studies have investigated the anti-AMI effects of allicin (Xu et al., 2020). Some studies have reported that sulfur compounds such as S-allylcysteine (SAC) and sodium hydrosulfide (NaHS) can protect against AMI by promoting hydrogen sulfide (H_2_S) production (Chuah et al., 2007; Bian et al., 2006). H_2_S has been shown to play a role in the regulation of Ca^2+^ homeostasis in cardiomyocytes (Peng et al., 2022; Wu et al., 2018). This prompts inquiry into whether allicin, similar to other sulfur-containing compounds, elicits an anti-AMI impact by regulating Ca^2+^ transport and intracellular Ca^2+^ homeostasis *via* H_2_S production enhancement.

In mammalian systems, the expression and function of three endogenous H_2_S synthetases—cystathionine β-synthase (CBS), cystathionine γ-lyase (CSE), and 3-mercaptopyruvate sulfurtransferase (3-MST)—in various tissues remain a subject of debate (Donnarumma et al., 2017). A previous study confirmed that the administration of DL-propargylglycine (PAG, a CSE inhibitor) in the absence of amino-oxyacetate acid (AOAA, a CBS inhibitor) significantly reduced but did not entirely abolish the protective effects of allicin on AMI injury by dilating coronary arteries and regulating calcium homeostasis in cardiomyocytes (Cui et al., 2022). These findings suggest that the anti-AMI effects of allicin are associated with H_2_S production, mediated not only through CSE but also *via* other enzymatic pathways. This study aimed to investigate the expression of hydrogen sulfide synthases in the cardiovascular system and assess the protective effects of allicin in an AMI rat model using hydrogen sulfide synthase inhibitors. The objective was to elucidate the relationship between allicin and H_2_S generation. It was hypothesized that allicin may regulate Ca^2+^ homeostasis by enhancing H_2_S production through multiple pathways, thereby providing protection against AMI injury.

## 2 Materials and methods

### 2.1 Reagents and antibodies

Allicin (a colorless or pale yellow clear liquid, sealed cryopreservation, 5.2 mg/mL) was biosynthesized with alliin and alliinase extracted from garlic at low temperature and was provided by Xinjiang Ailexin Pharmaceutical (the Xinjiang Uygur Autonomous Region, China). DL-propargylglycine (PAG), an inhibitor of the H_2_S synthetase cystathionine-gamma-lyase (CSE), was obtained from Shanghai Yuanye Bio-Technology (Yuanye Bio-Technology, Shanghai, China). Amino-oxyacetate acid (AOAA), an inhibitor of the H_2_S synthetase cystathionine-beta-synthase (CBS), was procured from LAMI Bio-Technology (LAMI Bio-Technology, Shanghai, China). Additionally, diltiazem, a Ca^2+^-channel blocker, was obtained from Tianjin Tanabe Pharmaceutical Co., Ltd. (Tanabe, Tianjin, China). All other reagents used were of analytical purity grade. All primary antibodies used in the experiments included GAPDH (UM4002, 1:2000 dilution, Utibody), CaMKII (ab134041, 1:1000 dilution, Abcam), phospho-CaMKII (ab171095, 1:1000 dilution, Abcam), SERCA2a (ab150435, 1:1000 dilution, Abcam), NCX-1 (ab177952, 1:1000 dilution, Abcam), RyR (ab219798, 1:1000 dilution, Abcam) and phospho-RyR2 (Ser2814) (AF2303, 1:1000 dilution, Affinity).

### 2.2 Experimental protocol

The experimental protocols utilized male Sprague Dawley (SD) rats aged six to 8 weeks and weighing 180–200 g. All animal procedures conformed to the guidelines from Directive 2010/63/EU of the European Parliament on the Protection of Animals Used for Scientific Plants or the National Institutes of Health (NIH) Guide for the Care and Use of Laboratory Animals and were approved by the Medical Ethics Committee of Xiyuan Hospital, China Academy of Chinese Medical Sciences (2024XLC093-2). General anesthesia was administered to the rats, followed by thoracotomy at the fourth intercostal space to expose the heart and left anterior descending coronary artery (LAD) for AMI induction. Specifically, rats were anesthetized with 1% pentobarbital sodium at 45 mg/kg body weight, intubated, and mechanically ventilated. Subsequently, upon left thoracotomy, the heart was exposed, and a 6–0 silk suture was utilized to permanently ligate the LAD about 2 mm below the left atrium. Confirmation of AMI occurrence was based on ST segment elevation on an electrocardiogram and bulging of the corresponding left ventricle segment. In the sham group, the same surgical procedure was performed, except only threading without ligation under the LAD branch of the coronary artery was conducted.

After the AMI model was established, the rats were divided into eight groups (*n* = 20 per group) on a random number table: the sham group, model group, diltiazem 8.1 mg/kg group, allicin 14 mg/kg group, allicin 7 mg/kg group, PAG (allicin 14 mg/kg + PAG 32 mg/kg) group, AOAA (allicin 14 mg/kg + AOAA 10 mg/kg) group, and PAG + AOAA (allicin 14 mg/kg + PAG 32 mg/kg + AOAA 10 mg/kg) group. All groups received intraperitoneal injections once a day for 7 days. As a result of acute heart failure occurring in some rats following surgery, which led to mortality, the final number of rats included in the study ranged from 16 to 19 per group. Notably, diltiazem, a calcium antagonist, is commonly used for the treatment of ischemic heart disease (Pearle, 1988; Chaffman and Brogden, 1985). The dosages of diltiazem, allicin, PAG, and AOAA were determined based on our previous studies as well as the relevant literature (Cui et al., 2020; Cui et al., 2022; El-Sayed et al., 2021).

### 2.3 Echocardiography

The rats underwent transthoracic echocardiography 7 days post-surgery using a Vevo 3100 echocardiography system (Visual Sonics Inc., Toronto, Canada) to assess cardiac function. The rats were anesthetized with 1.5%–2% isoflurane *via* continuous inhalation and placed on a heating pad (37°C) for warmth. Echocardiography (M-mode and B-mode imaging) was performed after applying ultrasound transmission gel to the chest. Measurements of the left ventricular (LV) internal diameter and thickness of the anterior wall were taken at end-diastole (LVID d, LVAW d) and end-systole (LVID s, LVAW s) during M-mode recordings. Left ventricular fractional shortening (FS), ejection fraction (EF), and stroke volume (SV) were calculated in a blinded manner for each rat.

### 2.4 Myocardial staining

The rats underwent intraperitoneal injection of 1% sodium pentobarbital solution at 45 mg/kg body weight for anesthesia and were euthanized by cervical dislocation. Subsequently, rat hearts were excised and sectioned into 1-mm thick slices across the left ventricular long-axis below the ligature. To identify the necrotic area, the heart slices were incubated with nitro-blue tetrazolium chloride (Sigma‒Aldrich, St. Louis, USA) for 3 min at 22 ± 2 °C. The necrotic area was quantified as a percentage of the ventricular or total area using Image-Pro Plus software (version 6.0, Media Cybernetics, Silver Springs, USA).

### 2.5 cTnI, LDH, and H_2_S measurements

Blood samples were collected from the abdominal aorta prior to sacrifice. The collected blood samples were maintained at 22 ± 2°C for 30 min before being centrifuged at 975.87 ×*g* for 10 min. The supernatants were then collected for the determination of serum cardiac troponin I (cTnI) and lactate dehydrogenase (LDH) levels. According to the manufacturer’s instructions, H_2_S levels in the serum and border zone of myocardial necrosis tissue were measured *via* methylene blue spectrophotometry at 665 nm (Nanjing Jiancheng Bioengineering Institute, Nanjing, China).

### 2.6 CSE and CBS measurements

Rat serum was prepared according to previously established methods. Levels of CSE and CBS in blood serum were assessed using enzyme-linked immunosorbent assay (ELISA) kits (Lanji Biotechnology Co., Ltd., Shanghai, China) as per the manufacturer’s instructions. The border zone of myocardial necrotic tissues and coronary arteries was fixed using 4% (v/v) paraformaldehyde, followed by a 30-min incubation with dimethyl benzene, and subsequent serum blocking for 60 min. Specimens were then exposed to CSE antibody (Proteintech, Wuhan, China) or CBS antibody (Proteintech, Wuhan, China) for 24 h at 4°C, followed by incubation with goat anti-rabbit IgG (H + L) fluorescein isothiocyanate-conjugated polyclonal antibody (Bai Aotong Experimental Materials Center, Luoyang, China) in the dark at 37°C for 60 min. Following washing with phosphate buffer solution, nuclei were stained with 4′,6-diamidino-2-phenylindole (St. Louis, MO, USA), and images were captured using an upright fluorescence microscope (DM-LFS, Leica, Germany) at ×400 magnification.

### 2.7 Histologic examination

The heart was harvested, weighed, washed in phosphate buffer, fixed in 4% paraformaldehyde overnight, and embedded in paraffin. Each paraffin-embedded heart was sectioned into 4-µm thick sections through the necrotic area, stained with hematoxylin and eosin (H&E), and observed for morphology using a stereomicroscope (Olympus SZ61, Tokyo, Japan).

### 2.8 Ca^2+^ transport in rat cardiomyocytes

Following the final administration, rats were anesthetized, and their hearts were swiftly excised, with cardiomyocytes from the junctional zone of myocardial necrosis promptly isolated. These isolated cardiomyocytes were then incubated with 2 µM Fura-2 a.m. (Sigma‒Aldrich, St. Louis, USA) in darkness at 22° ± 2°C for 30 min. Subsequently, cells were washed and resuspended twice in Tyrode’s solution (137.0 mM NaCl, 1.2 mM NaH_2_PO_4_, 5.0 mM KCl, 1.2 mM MgCl_2_, 10.0 mM HEPES, 10.0 mM glucose, 1.2 mM CaCl_2_, pH 7.4) before being placed in a cell chamber. The myocytes were then stimulated at a pacing frequency of 1 Hz with 4 ms electrical stimulation to induce contraction and exposed to excitation wavelengths of 340 or 380 nm. The emitted fluorescent signal was detected at 510 nm. Simultaneous recording of sarcomere length and fluorescence intensity (reflecting Ca^2+^ concentration) was performed using a cell contraction-ion detection system (IonOptix, Westwood, USA). Contractility parameters, including amplitude, peak time, systolic half-time of decay (T_50_), and diastolic T_50_, were assessed. Ca^2+^ transient parameters, such as amplitude, maximum ascending velocity, maximum descending velocity, and Ca^2+^ decline time constant, were also documented. Additionally, Tau _NCX_ and Tau _SERCA_, indicative of Ca^2+^ transient elimination time constants, as well as SR Ca^2+^ content (△F/F_0_) and Ca^2+^ leakage levels (△F_leak_/F_0_), were measured based on previously outlined methodologies (Cui et al., 2022).

### 2.9 Western blot

Myocardial tissue in the junctional zone of myocardial necrosis in rats was collected, homogenized, and lysed. Protein samples were prepared following the manufacturer’s protocol for gel electrophoresis (NuPAGE 4%–12% Bis-Tris, Invitrogen, Carlsbad, USA). A normalized final loading concentration of 10–30 µg per well was used for all experiments. The proteins were then transferred to a polyvinylidene fluoride membrane (Bio-Rad, Hercules, USA) for immunoblotting using the designated antibodies.

### 2.10 Statistical analysis

The data are expressed as the mean ± standard deviation (SD). The distribution of the data was assessed using the Shapiro‒Wilk normality test. One-way ANOVA followed by Tukey’s honestly significant difference (HSD) *post hoc* test or Tamhane’s *post hoc* test was used to determine differences between groups for normally distributed data. For data that were not normally distributed, the Kruskal‒Wallis test was used. All the statistical analyses were performed using SPSS statistical software (SPSS 26.0, IBM, Armonk, NY), and all the histograms were created using GraphPad Prism 9.0 software (GraphPad Software, San Diego, USA). A P value < 0.05 was considered to indicate statistical significance.

## 3 Results

### 3.1 Allicin improves cardiac function in AMI rats

**FIGURE 1:**
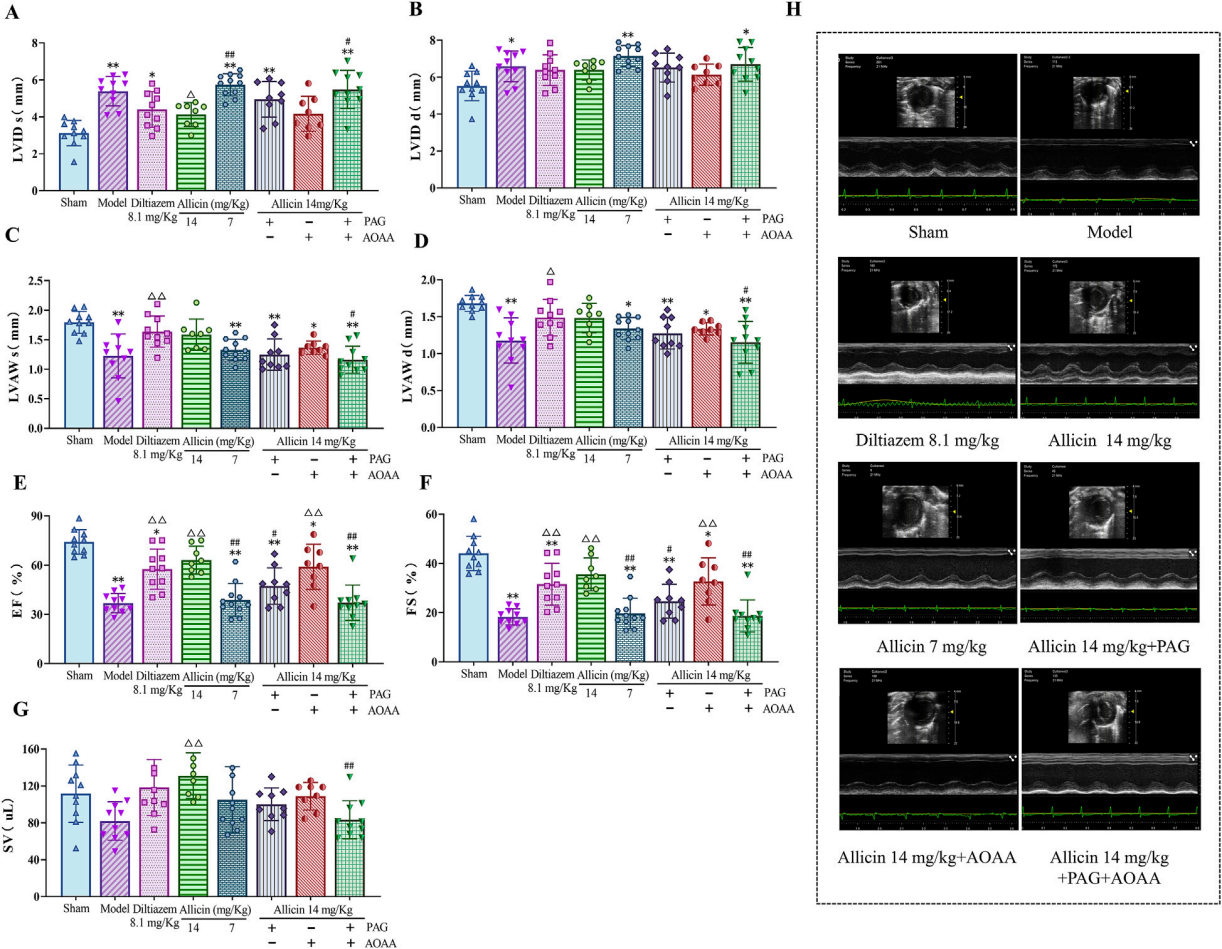
Allicin improved cardiac function in acute myocardial infarction model rats. **(A‒G)** Statistical scatter plots of the left ventricular (LV) internal diameter and thickness of the anterior wall at end-systole (LVID s, LVAW s) and end-diastole (LVID d, LVAW d), ejection fraction (EF), fractional shortening (FS), and stroke volume (SV) in the different groups. **(H)** Representative images of ultrasonic function in each group. Statistical analysis was performed by one-way ANOVA and Tukey’s honestly significant difference (HSD) *post hoc* test, and the data are expressed as the mean ± SD (*n* = 8–11). *p < 0.05, **p < 0.01 vs. Sham; ^△^ *p* < 0.05, ^△△^ *p* < 0.01 vs. Model; ^#^ *p* < 0.05, ^##^ *p* < 0.01 vs. 14 mg/kg allicin.

To assess the cardioprotective effects of allicin on AMI, we initially utilized small animal echocardiography to evaluate cardiac function in AMI model rats. The echocardiographic results showed that compared to those in the sham group, the LVID s and LVID d were significantly greater, and the LVAW s, LVAW d, EF and FS were significantly lower in the model group. Compared to those in the model group, the LVID s were significantly lower and LVAW s, LVAW d, EF, FS, and SV were significantly greater in the diltiazem 8.1 mg/kg and allicin 14 mg/kg groups. The strength of the effect of allicin in the 14 mg/kg group was comparable to that in the 8.1 mg/kg diltiazem group. After PAG alone or combined with PAG and AOAA, the above effects of allicin were significantly weakened, and there was no significant difference between the two groups, while AOAA alone had no significant weakening effect (Figure 1 **)**. Hence, allicin significantly improved the cardiac function of AMI rats, which was mainly mediated by CSE.

### 3.2 Allicin reduces myocardial necrotic size and myocardial enzyme levels in AMI rats

**FIGURE 2:**
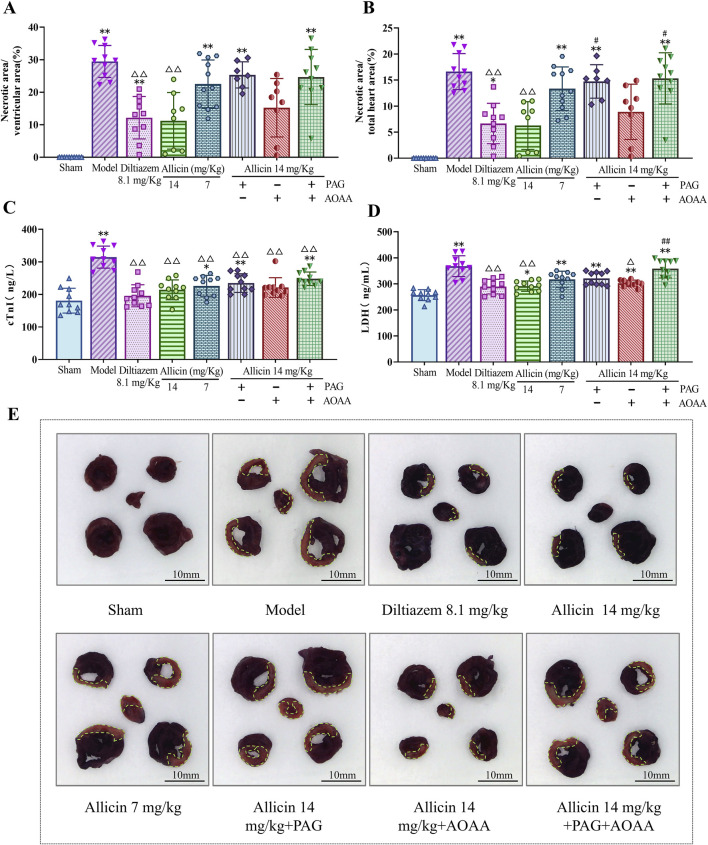
Allicin reduced the myocardial necrosis area and serum myocardial enzyme levels in acute myocardial infarction rats. **(A,B)** Percentage of myocardial necrosis area relative to the ventricular and total heart areas, respectively. **(C,D)** Myocardial enzyme (cTnI, LDH) levels in the serum of acute myocardial infarction rats. **(E)** Representative myocardial necrosis areas in each group; scale bar = 10 mm. The area circled in green represents the area of myocardial necrosis. Statistical analysis was performed by one-way ANOVA, Tukey’s honestly significant difference (HSD) *post hoc* test and Tamhane *post hoc* test, and the data are expressed as the means ±SDs (*n* = 7–11). *p < 0.05, **p < 0.01 vs. Sham; ^△^ *p* < 0.05, ^△△^ *p* < 0.01 vs. Model; ^#^ *p* < 0.05, ^##^ *p* < 0.01 vs. 14 mg/kg allicin.

To further evaluate the anti-AMI effects of allicin, we measured myocardial necrotic areas and myocardial enzyme levels in AMI rats. Compared to those in the sham group, the percentages of myocardial necrosis in the ventricular area and total heart area, as well as the levels of cTnI and LDH, were significantly greater in the model group. Compared to those in the model group, myocardial necrosis as a percentage of the ventricular area and total heart area and LDH and cTnI levels were significantly lower in the 8.1 mg/kg diltiazem and 14 mg/kg allicin groups. The strength of the effect of allicin in the 14 mg/kg group was comparable to that in the 8.1 mg/kg diltiazem group. After PAG treatment alone or combined with PAG and AOAA, allicin significantly decreased the percentage of myocardial necrosis in the total heart area and the LDH level, while AOAA treatment alone did not significantly decrease the percentage of myocardial necrosis (Figure 2 **)**. Therefore, allicin significantly reduced myocardial necrosis size and myocardial enzyme levels in AMI rats, and these effects were significantly weakened by PAG alone or in combination with AOAA.

### 3.3 Allicin increases H_2_S level in AMI rats

**FIGURE 3:**
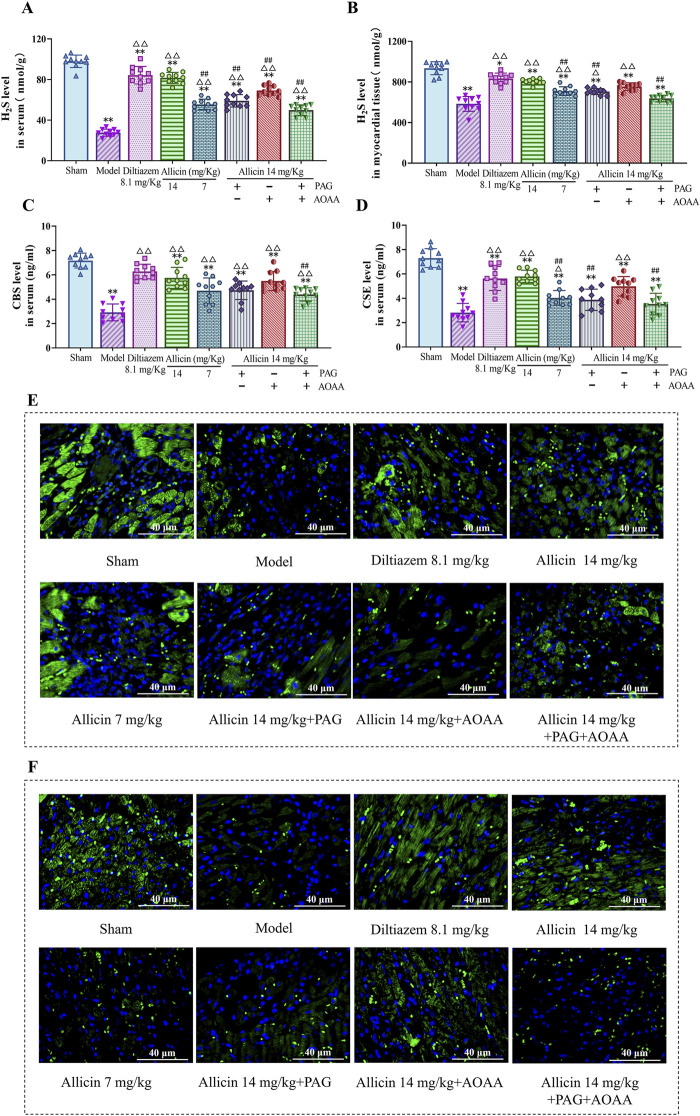
Allicin increased H_2_S levels and H_2_S synthase (CBS, CSE) in myocardial tissue and serum. **(A,B)** H_2_S levels in the serum and myocardial tissues of rats in each group. **(C,D)** H_2_S synthase (CBS, CSE**)** levels in the serum of rats in each group. **(E,F)** Fluorescence images of representative CBS and CSE in myocardial tissue from each group. Scale bar = 40 µm. Statistical analysis was performed by one-way ANOVA, Tukey’s honestly significant difference (HSD) *post hoc* test and Tamhane *post hoc* test, and the data are expressed as the means ±SDs (*n* = 10). *p < 0.05, **p < 0.01 vs. Sham; ^△^ *p* < 0.05, ^△△^ *p* < 0.01 vs. Model; ^##^ *p* < 0.01 vs. 14 mg/kg allicin.

Subsequently, to examine the relationship between the anti-AMI effects of allicin and H_2_S production, we measured the expression of H_2_S level in the serum and myocardial tissues of the rats. The results demonstrated significant decreases in H_2_S level in serum and myocardial tissues, in the model group compared to those in the sham group. However, treatment with 14 mg/kg allicin and 8.1 mg/kg diltiazem significantly increased the H_2_S level in both serum and myocardial tissues. Importantly, the effect of allicin in the 14 mg/kg group was comparable to that in the 8.1 mg/kg diltiazem group. Notably, compared to the 14 mg/kg allicin group, the H_2_S level was significantly lower in the PAG, AOAA, and PAG + AOAA groups. Consequently, allicin notably elevated both H_2_S level in the serum and myocardial tissues of AMI rats (Figures 3A,B). In addition, we were surprised to find that allicin significantly upregulated the levels of H_2_S synthase CBS and CSE in serum and myocardial tissue of AMI rats (Figures 3C–F). Therefore, allicin effectively boosted H_2_S production in AMI rats, offering a potential mechanism for its cardioprotective effects.

### 3.4 Allicin ameliorates myocardial histopathological changes in AMI rats

**FIGURE 4:**
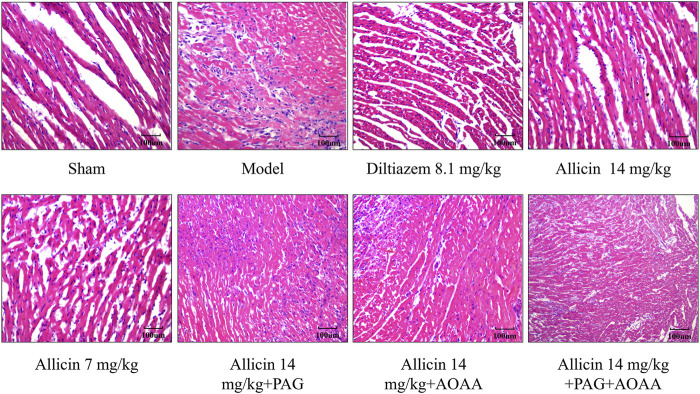
Allicin improved the pathological morphology of the myocardium in acute myocardial infarction rats. H&E staining, magnification ×200. Scale bar = 100 µm (*n* = 4).

The myocardial cells displayed uniform staining with HE, and the striated filaments of the cardiac myocytes were neatly organized with distinct cell boundaries. In the sham group, typical morphology of normal cardiomyocytes was observed, showing no signs of degeneration, necrosis, hemorrhage, or inflammatory cell infiltration. However, in the model group, the normal cell structure was disrupted, leading to extensive inflammatory cell infiltration and tissue edema, indicative of a typical heart attack. However, compared to those in the model group, the inflammatory cell infiltration in the allicin 14 mg/kg and diltiazem 8.1 mg/kg groups decreased, the arrangement of cardiomyocytes was slightly disrupted, and the myocardial tissue was slightly edema. Compared with those in the allicin 14 mg/kg group, the PAG alone, AOAA alone, and combined PAG and AOAA groups all exhibited exacerbated pathological damage (Figure 4 **)**. In conclusion, allicin effectively improved the histopathological morphology of the myocardium in rats with AMI, and this effect was weakened by PAG and AOAA treatment.

### 3.5 Allicin regulates cardiomyocyte contraction kinetics and cardiomyocyte Ca^2+^ transient amplitude in AMI rats

**FIGURE 5:**
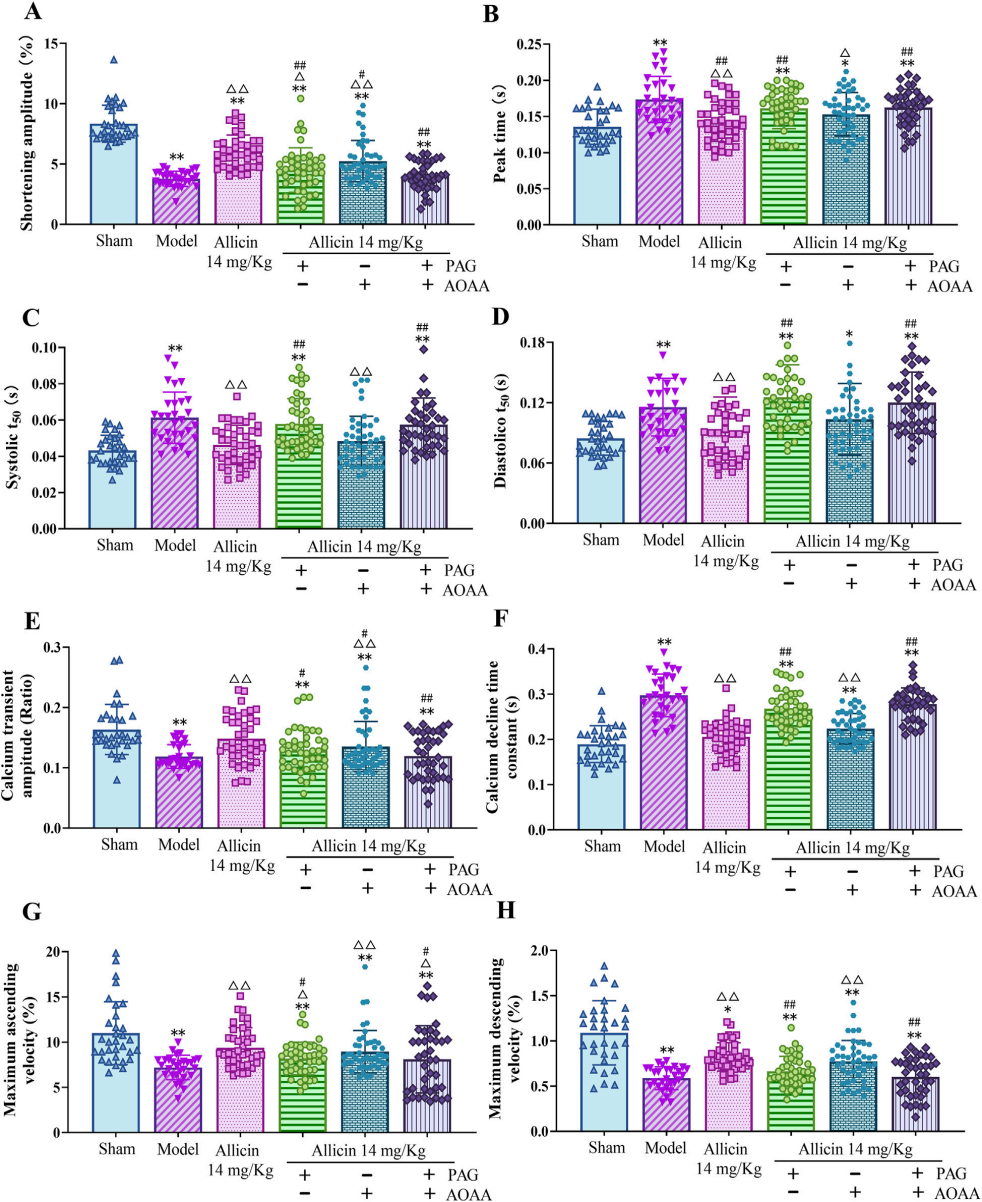
Allicin regulated cardiomyocyte contraction kinetics and cardiomyocyte calcium transient amplitude in acute myocardial infarction rats. **(A)** Shortening amplitude; **(B)** Peak time; **(C)** Systolic T_50_; **(D)** Diastolic T_50_; **(E)** Calcium transient amplitude; **(F)** calcium decline time constant; **(G)** Maximum ascending velocity; **(H)** Maximum descending velocity. Statistical analysis was performed by the Kruskal‒Wallis test, and the data are presented as the mean ± SD (*n* = 29–46 cardiomyocytes from four rats). *p < 0.05, **p < 0.01 vs. Sham; ^△^ *p* < 0.05, ^△△^ *p* < 0.01 vs. Model; ^#^ *p* < 0.05, ^##^ *p* < 0.01 vs. 14 mg/kg allicin. Abbreviations: T_50_, half-life of decay.

Alterations in the Ca^2+^ concentration in cardiomyocytes are a major cause of cardiomyocyte damage following AMI. Therefore, we used cardiomyocyte contraction kinetics and cytoplasmic Ca^2+^ transient assays to assess the role of allicin in the regulation of Ca^2+^ transport in cardiomyocytes from AMI rats. Compared to those in the sham group, the contraction amplitude, Ca^2+^ transient amplitude, maximum ascending velocity and maximum descending velocity were significantly lower, and the peak time, systolic T_50_, diastolic T_50_, and Ca^2+^ decline time constant were significantly greater in the model group. After treatment with 14 mg/kg allicin, the above effects were significantly improved. After PAG treatment alone or combined with PAG and AOAA treatment, the above improvement effects of allicin were significantly weakened but not completely eliminated, while AOAA treatment alone significantly weakened the improvements in contraction amplitude and Ca^2+^ transient amplitude caused by allicin but not completely eliminated (Figure 5). Therefore, allicin can significantly regulate cardiomyocyte contraction dynamics, which can be significantly weakened but not completely blocked by PAG alone or combined with PAG and AOAA.

### 3.6 Allicin improves SERCA-dependent Ca^2+^ reuptake and NCX-dependent Ca^2+^ removal in cardiomyocytes from AMI rats

**FIGURE 6:**
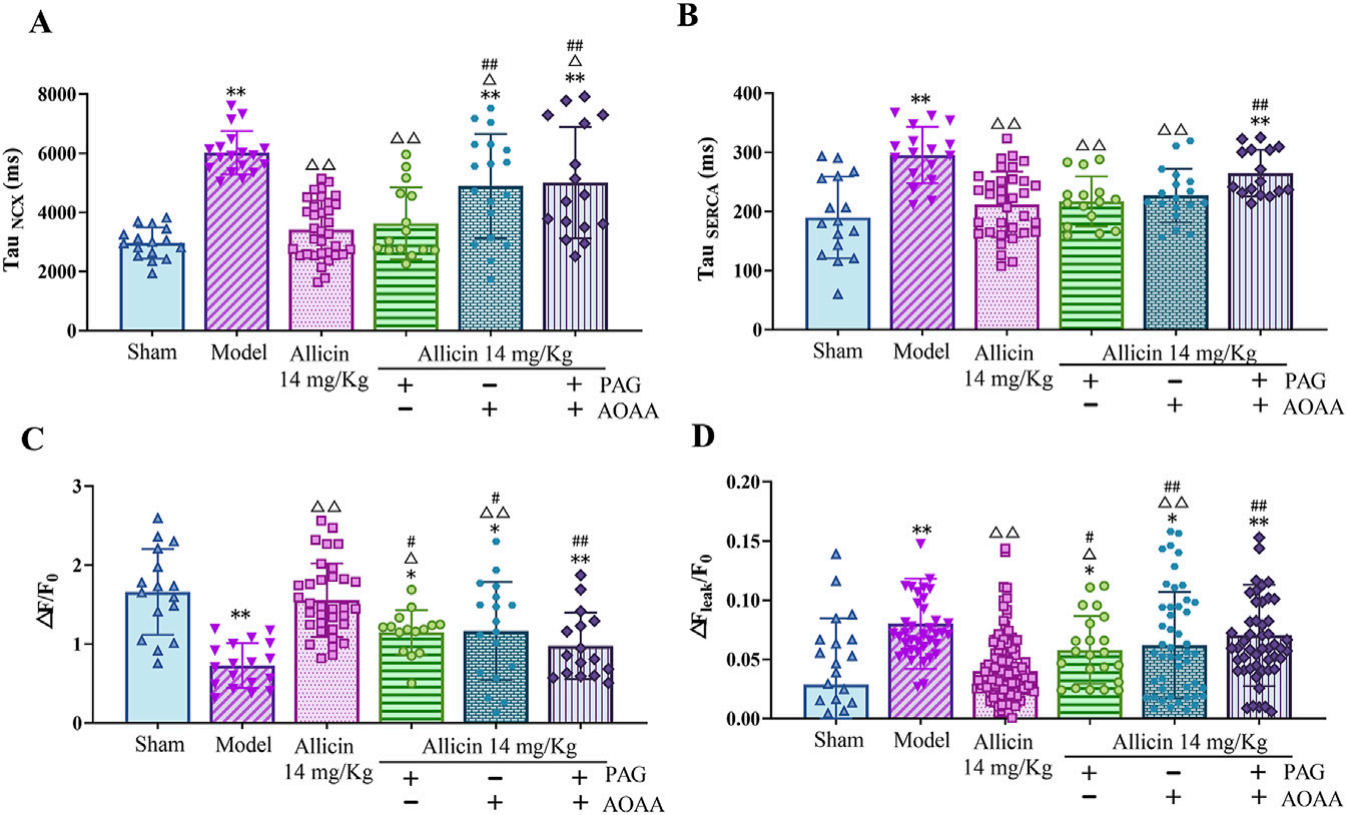
Allicin regulated calcium transport in cardiomyocytes from acute myocardial infarction rats. **(A)** NCX calcium removal function, Tau_NCX_ (*n* = 15–34 cardiomyocytes from four rats); **(B)** SERCA calcium reuptake function, Tau_SERCA_ (*n* = 15–34 cardiomyocytes from four rats); **(C)** Sarcoplasmic reticulum calcium content, △F/F_0_ (*n* = 15–34 cardiomyocytes from four rats); **(D)** Calcium leakage levels, △F_leak_/F_0_ (*n* = 22–123 cardiomyocytes from four rats). Statistical analysis was performed by the Kruskal‒Wallis test, and the data are presented as the means ±SDs. ^*^ *p* < 0.05, ^**^ *p* < 0.01 vs. Sham; ^△^ *p* < 0.05, ^△△^ *p* < 0.01 vs. Model; ^#^ *p* < 0.05, ^##^ *p* < 0.01 vs. 14 mg/kg allicin.

SERCA-mediated Ca^2+^ reuptake into the sarcoplasmic reticulum (SR) and NCX-dependent intracellular Ca^2+^ efflux play crucial roles in reducing the intracellular Ca^2+^ concentration and promoting cellular diastole in cardiomyocytes. The results showed that the levels of the Tau _NCX_ and the Tau _SERCA_ were significantly greater in the model group than in the sham group, suggesting that the functions of the NCX and SERCA are attenuated after AMI. In addition, the levels of the Tau _NCX_ and the Tau _SERCA_ in the 14 mg/kg allicin group were significantly lower than those in the model group. After the addition of PAG alone, no significant changes in the levels of the Tau _NCX_ or the Tau _SERCA_ were observed. However, compared to those in the 14 mg/kg allicin group, after the application of AOAA alone, the level of the Tau _NCX_ was significantly greater, and after the combined application of PAG + AOAA, the levels of the Tau _NCX_ and the Tau _SERCA_ were both significantly greater (Figures 6A,B). Thus, allicin can effectively improve SERCA and NCX functions following AMI. Although these effects can be significantly weakened, they are not completely blocked by AOAA alone or PAG and AOAA combined.

### 3.7 Allicin regulates the SR Ca^2+^ content and Ca^2+^ leakage in cardiomyocytes from AMI rats

Next, we measured the SR Ca^2+^ content and Ca^2+^ leakage in cardiomyocytes. The SR Ca^2+^ content of myocardial cells (△F/F_0_) was significantly lower in the model group than in the sham group, while 14 mg/kg allicin significantly increased the SR Ca^2+^ content in the model group. In addition, the SR Ca^2+^ content was significantly lower after the addition of PAG, AOAA, or the combination of PAG + AOAA than after the addition of 14 mg/kg allicin. In addition, compared with that in the sham group, Ca^2+^ leakage (△F_leak_/F_0_) was significantly greater in the model group, whereas 14 mg/kg allicin significantly decreased Ca^2+^ leakage compared to that in the model group. Moreover, compared with that in the 14 mg/kg allicin group, Ca^2+^ leakage was significantly greater in the PAG alone, AOAA alone, and combined PAG and AOAA groups (Figures 6C,D). These findings indicate that allicin effectively ameliorates the decrease in the SR Ca^2+^ content and the increase in Ca^2+^ leakage following AMI. However, these beneficial effects of allicin can be significantly attenuated, but not completely blocked, by PAG alone, AOAA alone, or PAG and AOAA combined treatment.

### 3.8 Allicin regulates the expression of key proteins involved in Ca^2+^ transport in cardiomyocytes from AMI rats

**FIGURE 7:**
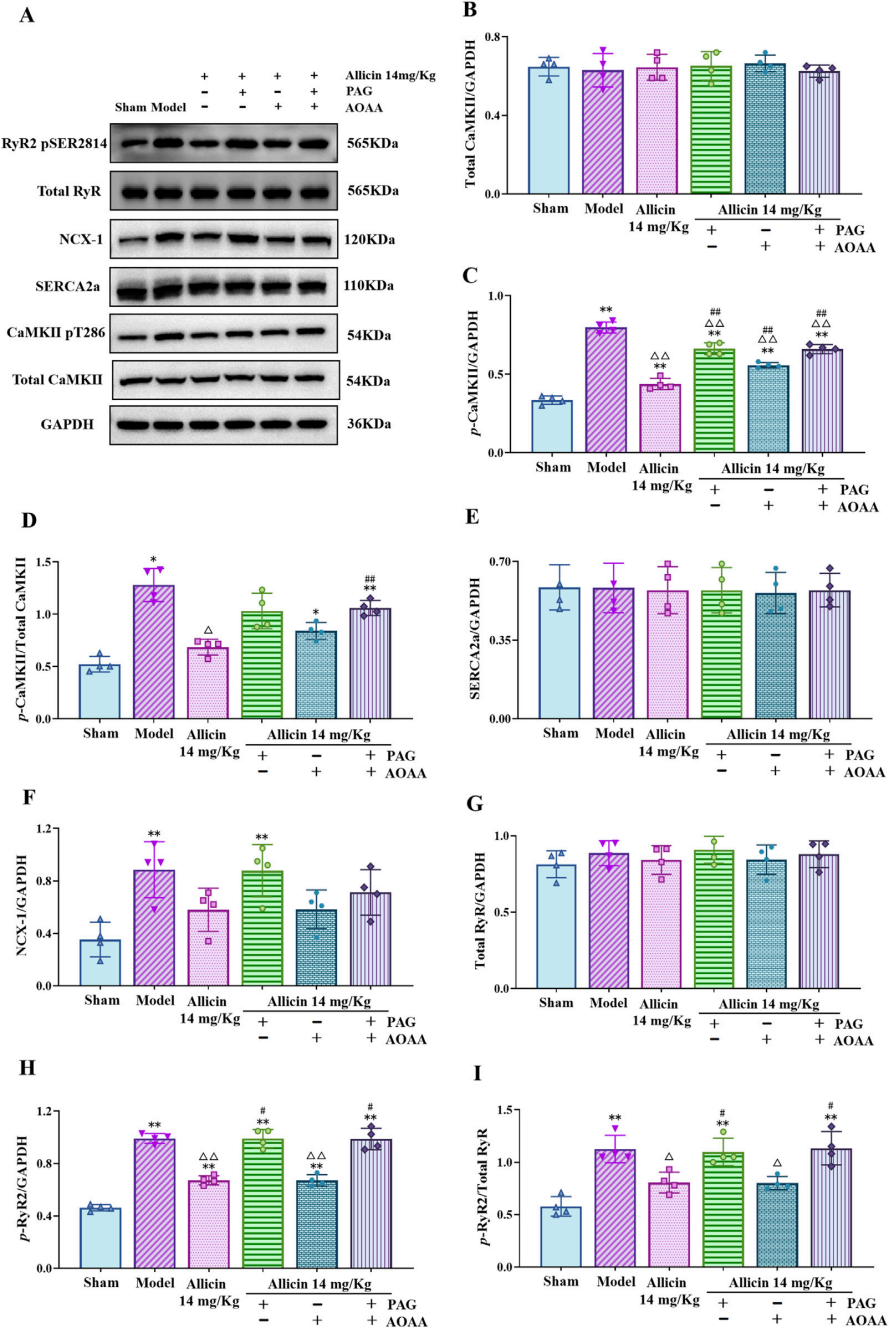
Allicin regulated the expression of key calcium transport proteins in the myocardial tissues of acute myocardial infarction rats. **(A)** Representative immunoblots of myocardial tissues for p-RyR2, total RyR, NCX-1, SERCA2a, p-CaMKII, CaMKII and GAPDH obtained from rats. **(B–I)** Quantitative analyses of total CaMKII, p-CaMKII, SERCA2a, NCX-1, total RyR, p-RyR2 normalized to GAPDH, the ratio of p-CaMKII/total CaMKII **(D)** and p-RyR2/total RyR **(I)**. Statistical analysis was performed by one-way ANOVA, Tukey’s honestly significant difference (HSD) *post hoc* test and Tamhane *post hoc* test, and the data are presented as the means ±SDs (*n* = 4). *p < 0.05, **p < 0.01 vs. Sham; ^△^ *p* < 0.05, ^△△^ *p* < 0.01 vs. Model; ^#^ *p* < 0.05, ^##^ *p* < 0.01 vs. 14 mg/kg allicin.

After AMI, the expression of key Ca^2+^ transport proteins in cardiomyocytes also changed. The Western blot results showed that the expression of p-CaMKII, p-RyR2, NCX-1, the p-CaMKII/total CaMKII ratio and the p-RyR2/total RyR ratio were significantly greater and that the expression of SERCA2a was no significant difference in the model group than in the sham group. The expression of p-CaMKII and p-RyR2 and the ratio of p-CaMKII/total CaMKII and p-RyR2/total RyR were significantly decreased after the application of 14 mg/kg allicin. Moreover, the p-CaMKII/total CaMKII ratio can be increased by the combination of PAG + AOAA. The ratio of p-RyR2/total RyR was significantly increased by the application of PAG alone or the combination of PAG + AOAA (Figure 7). These results confirm the regulatory effects of allicin on Ca^2+^ transport to some extent. Overall, allicin effectively regulates the expression of key proteins involved in Ca^2+^ transport in cardiomyocytes following AMI.

## 4 Discussion

AMI is a major cause of morbidity and mortality worldwide, driven by the increasing aging population. Its occurrence is related mainly to risk factors such as overwork, long-term smoking, heavy alcohol consumption, hypertension, and hyperlipidemia (Frangogiannis, 2015). PCI is the first-line treatment for AMI patients and can restore the myocardial blood supply quickly and effectively, reduce myocardial necrosis, and increase the survival rate. However, not all AMI patients are suitable for immediate PCI. For these patients, medication must be given first to temporarily maintain vital signs. For example, when AMI patients suffer from severe shock, heart failure, severe electrolyte disturbance, or infection, medication must be applied to relieve the condition to buy time until PCI can be safely performed. Moreover, medication is a preferable option for elderly patients, those with multiple comorbidities, or those at high risk for surgery compared to PCI. Currently, medical treatment comprises Western medicine and traditional Chinese medicine. The use of Western medicine, such as antiplatelet drugs (e.g., aspirin), antianginal drugs (e.g., nitroglycerin), and anticoagulant drugs (e.g., heparin), can alleviate the clinical symptoms of patients to some extent. However, adverse drug reactions, such as gastrointestinal discomfort from aspirin, hypotension from nitroglycerin, and bleeding from heparin, should not be overlooked. In contrast, traditional Chinese medicine offers advantages such as multiple therapeutic effects, multiple targets, and minimal toxic and side effects. Therefore, the treatment of AMI using traditional Chinese medicine has become a focus of research.

As a food source and medicinal plant, garlic is a traditional Chinese medicine that has been used in China and Southeast Asia for thousands of years (Khatua et al., 2013). Garlic has beneficial effects on a wide spectrum of diseases, including cancer, diabetes, microbial infections, and immunological disorders. Recent studies have shown that garlic and its active ingredients have potential therapeutic effects on cardiovascular diseases, including arrhythmia (Sungnoon et al., 2008; Fattahi et al., 2013), hypertrophy (Bradley et al., 2016; Zhu et al., 2018; Mocayar et al., 2020), hypertension (Cui et al., 2020), hyperlipidemia (Chan et al., 2013), and atherosclerosis (Lei et al., 2010). However, their protective effects on AMI have rarely been studied, and only a few studies are available (Xu et al., 2020). We previously demonstrated that allicin, the main active ingredient of garlic, plays an antiapoptotic and antifibrotic role in myocardial ischemia (Cui et al., 2022; Ma et al., 2017). Thus, the present study aimed to further determine the protective effects of allicin on AMI and explore the underlying mechanisms involved. The results showed that allicin exerted significant anti-AMI effects on the AMI rat model, as evidenced by reduced myocardial necrosis, improved heart function, reduced myocardial enzyme levels, and improved pathological morphology of myocardial tissue.

Because of limited research on the anti-AMI effect of allicin, there is even less evidence regarding the associated mechanisms. Only a few available studies have shown that allicin exerts its anti-AMI effects by modulating the JNK signaling pathway and inhibiting cardiomyocyte apoptosis induced by Ca^2+^ overload through the PI3K/GRK2/PLC-γ/IP3R signaling pathway (Gao et al., 2021). Numerous studies have indicated that Ca^2+^ overload in cardiomyocytes is a critical mechanism for myocardial cell damage resulting from myocardial ischemia and adverse stimulation (Cui et al., 2022; Song et al., 2022; Zhao et al., 2016). This overload not only compromises the contractile function of cardiomyocytes but also disrupts mitochondrial energy metabolism and morphology, ultimately leading to cardiomyocyte death and severe cardiac dysfunction (Bulluck et al., 2016; Wollert and Drexler, 2010). Indeed, Ca^2+^ concentration in cardiomyocytes is influenced and regulated by a series of Ca^2+^ transport activities. During normal excitation-contraction coupling in cardiomyocytes, cell excitation initiates the opening of voltage-dependent L-type Ca^2+^ channels on the cell membrane, causing the influx of extracellular Ca^2+^ and the activation of RyRs on the SR. These RyRs, particularly the RyR2 subtype predominant in myocardial tissue, facilitate the release of Ca^2+^ from the SR to the cytosol. Following RyR2 activation, the SR rapidly discharges a large quantity of Ca^2+^, leading to a transient and significant rise in cytoplasmic Ca^2+^ concentration, which binds to myofilament proteins and initiates cell contraction. Conversely, decreased Ca^2+^ concentration results in cardiomyocyte dilation. In most mammals, approximately 70% of the reduction in intracellular Ca^2+^ concentration is ascribed to the reuptake of SERCA2a, a Ca^2+^ pump primarily expressed in the heart and situated in the SR (Zhihao et al., 2020). The remaining 30% reduction is mainly due to the removal of NCX, another Ca^2+^ pump located on the cell membrane, while less than 1% is associated with other mechanisms (Laver, 2007; MacLeod, 2016). Notably, NCX has three isoforms (NCX1-3) in mammals. Among the three isoforms, NCX-1 plays a crucial role in cardiac contractile activity and is the subject of extensive study (Xue et al., 2023). Under physiological conditions, NCX expels one Ca^2+^ out of the cell while simultaneously transporting three sodium ions (Na^+^) into the cell. These functions are regulated by various key proteins, with CaMKII being one of the most extensively studied upstream regulators of Ca^2+^ transport in cardiomyocytes. The CaMK family comprises CaMK I, II, IV, and K (Wayman et al., 2011), with CaMKII primarily engaged in cardiomyocyte Ca^2+^ transport (Reyes Gaido et al., 2023). On the one hand, CaMKII promotes the release of Ca^2+^ from the SR during the systolic period and facilitates SR Ca^2+^ leakage during the diastolic period by phosphorylating RyR2 (Wei et al., 2023), ultimately leading to reduced SR Ca^2+^ capacity and impaired cardiomyocyte contractile function. Conversely, CaMKII regulates SERCA2a activity by facilitates the phosphorylation of its downstream protein phospholamban (PLB), consequently contributing to the preservation of cardiomyocyte contraction function by enhancing Ca^2+^ reuptake and augmenting the Ca^2+^ storage capacity of the SR (Hu et al., 2020). CaMKII has two diametrically opposite pharmacological effects on activating RyR2 and SERCA2a. At present, most scholars believe that CaMKII-mediated activation of RyR2 rather than SERCA2a plays a dominant role under pathological conditions (Mattiazzi and Kranias, 2014).

In this study, we measured cardiomyocyte systolic dynamics, the amplitude and velocity of Ca^2+^ concentration changes, the Ca^2+^ capacity of the SR, the Ca^2+^ leakage level of the SR, the removal velocity of the NCX and the reuptake velocity of the SERCA. We also utilized Western blotting to assess the expression of key Ca^2+^ transport proteins. After AMI, the amplitude of cardiomyocyte contraction decreased, the amplitude and velocity of Ca^2+^ concentration changes decreased, the Ca^2+^ capacity of the SR decreased, the Ca^2+^ leakage level of the SR increased, the reuptake of SERCA decreased, Ca^2+^ removal of the NCX decreased, the expression of p-CaMKII, p-RyR2, and NCX-1 increased. After the administration of allicin, all of the above indicators improved significantly. These results suggested that Ca^2+^ transport disturbance and abnormal contractile function of cardiomyocytes are significant pathological characteristics of AMI and are caused by decreased Ca^2+^ efflux, decreased Ca^2+^ pool reuptake, decreased Ca^2+^ pool capacity, and increased Ca^2+^ leakage after AMI. Allicin protects against AMI by correcting the disturbance of Ca^2+^ transport. Furthermore, a puzzling phenomenon was observed in the present study; that is, Tau_NCX_ was significantly elevated in the model group compared with the sham group, while the expression of NCX-1 was significantly upregulated according to the Western blot results. An increase in the Tau_NCX_ indicates that NCX needs a longer time to excrete Ca^2+^ to regulate the intracellular Ca^2+^ concentration; in other words, the efficiency of NCX excreting Ca^2+^ from the cell is reduced. This phenomenon seems paradoxical with the increased expression of NCX-1. In fact, NCX is a bidirectional transporter with two modes of operation: Ca^2+^ efflux and Ca^2+^ influx. Under physiological conditions, NCX primarily operates in Ca^2+^-efflux mode, pumping one Ca^2+^ out of the cell while transporting three Na^+^ ions into the cell. Conversely, under pathological conditions, NCX switches to a Ca^2+^-influx mode, pumping one Ca^2+^ into the cell and three Na^+^ ions out of the cell (Valentim et al., 2022; Rose et al., 2020). Thus, our above confusion may be explained by the following: under AMI conditions, the compensatory increase in NCX-1 expression due to the decrease in Ca^2+^ reuptake by SERCA, but NCX activity is dominated by Ca^2+^ influx. Therefore, the Ca^2+^ efflux function of NCX was greatly weakened, and Ca^2+^ removal from the cell took longer, which was reflected by the increase in Tau_NCX_. The above speculation is supported by other studies; that is, both the expression of NCX-1 and the activity of NCX in Ca^2+^-influx mode are stronger under pathological conditions such as ischemia/reperfusion injury, arrhythmia, and heart failure (Matsumoto et al., 2003; Pott et al., 2011).

It is widely recognized that the consumption of raw garlic leads to an unpleasant odor in the oral cavity due to the presence of H_2_S. Previous research has indicated that other sulfur-containing compounds in garlic, such as diallyl disulfide (DADS) and S-allylcysteine (SAC), exhibit cardiovascular activities, including anti-myocardial ischemia and blood pressure reduction, by promoting H_2_S production (Bradley et al., 2016; Wen et al., 2018). Additionally, H_2_S and H_2_S donors, such as sodium hydrosulfide (NaHS) and sodium sulfide (Na_2_S), exhibit definite cardiovascular effects, including blood pressure reduction, atherosclerosis prevention, alleviation of pulmonary hypertension, and anti-myocardial ischemia (Cui et al., 2020; Li et al., 2019; Zhang et al., 2020). Consequently, we hypothesize that allicin, similar to other sulfur-containing compounds, can induce H_2_S production *in vivo*; thus, inhibiting myocardial ischemia through the regulation of Ca^2+^ transport. To substantiate this hypothesis, we observed whether the effects of allicin are decreased or eliminated when H_2_S production is inhibited by H_2_S synthase inhibitors. In mammalian systems, endogenous H_2_S production is primarily facilitated by three H_2_S synthases, namely, cystathionine β-synthase (CBS), cystathionine γ-lyase (CSE), and 3-mercaptopyruvate sulfurtransferase (3-MST) (Li et al., 2016). However, there is debate surrounding the tissue specificity of these three enzymes. For example, it is widely believed by most scholars that CSE is primarily expressed in the mammalian cardiovascular and respiratory systems, particularly in the vascular endothelium and cardiomyocytes (De Luca et al., 1974; Zhao et al., 2001). However, certain studies have demonstrated that CSE is also highly expressed in the liver, kidney, uterus, placenta, and pancreatic islets (Zhao et al., 2001; Yang et al., 2004). Regarding CBS, most scholars contend that it is primarily expressed in the central nervous system (Wang, 2012; Enokido et al., 2005), yet a few studies have indicated that it is highly expressed in cardiac tissues, liver, and kidney (Talaei et al., 2011). 3-MST is widely recognized to be expressed in all types of mammalian cells and tissues (Kimura, 2017), and is highly expressed in the brain, liver, kidney, testis, large intestine, and endocrine organs (Tomita et al., 2016). To determine the expression of H_2_S synthases in rat cardiac tissue, the levels of these three enzymes were evaluated through immunofluorescence staining in healthy rat myocardial tissue. The results revealed high expression of CSE and CBS, while 3-MST was scarcely expressed in the myocardial tissues of healthy rats (see Supplementary Figure S1). Consequently, we employed corresponding inhibitors of CSE and CBS to inhibit H_2_S production in this study. The results showed that the protective effects on AMI and the correction of Ca^2+^ transport disturbance caused by allicin were significantly weakened but not completely eliminated by the combination of a CSE inhibitor and a CBS inhibitor, suggesting that the mechanisms of allicin’s protective effects were related to H_2_S production, but there were other mechanisms unrelated to H_2_S. Importantly, the CSE inhibitor alone also significantly diminished the protective effects of allicin, while the CBS inhibitor alone did not significantly inhibit the protective effects of allicin. Furthermore, the strength of action of CSE inhibitors alone was equivalent to that of the combination of CSE inhibitor and CBS inhibitor. These findings indicate that allicin-mediated H_2_S generation is primarily mediated by CSE rather than CBS. Intriguingly, the present study revealed another noteworthy phenomenon: allicin not only elevates the levels of H_2_S in serum and myocardial tissues but also increases the levels of CSE and CBS, indicating that allicin not only acts as a direct H_2_S donor but also promotes H_2_S production by upregulating the levels of H_2_S synthases. Consequently, several questions arise. First, why does the AMI-protective effect of allicin remain largely intact when a CBS inhibitor alone is administered, despite the abundant expression of CBS in rat myocardial tissue? Furthermore, why is the protective impact of allicin not weakened by the CBS inhibitor alone, despite the inhibition of H_2_S production mediated by CBS, as indicated by reduced H_2_S levels in both serum and myocardial tissue? To some extent, a previously reported discovery may provide insight into these puzzling questions. An established CBS inhibitor known as AOAA (Zuhra et al., 2020; Petrosino et al., 2022), applied in this study, is recognized for its anti-inflammatory properties, as it inhibits the NLRP3-Caspase/IL-1β pathway. Consequently, this diminishes the area of myocardial infarction and enhances cardiac function in an AMI mouse model (Zhao et al., 2020). This revelation aids in understanding the aforementioned puzzling questions to a certain degree. Additionally, allicin can enhance H_2_S production either directly as a donor or indirectly by upregulating the expression of H_2_S synthases, CSE and CBS. However, the more effective pathway for promoting H_2_S production remains unclear. The specific mechanism by which allicin regulates CSE and CBS expression also requires further investigation. Future studies utilizing gene editing technology may provide deeper insights into these mechanisms and help clarify the precise role of allicin in H_2_S synthesis regulation.

## 5 Conclusion

In conclusion, our findings demonstrate that the anti-AMI effect of allicin can be attributed to its ability to regulate Ca^2+^ homeostasis in cardiomyocytes by promoting H_2_S generation. Therefore, the promotion of Ca^2+^ homeostasis and H_2_S production in cardiomyocytes may serve as a crucial therapeutic target for AMI, providing a novel direction for the treatment of AMI and other cardiovascular diseases associated with Ca^2+^ overload.

## Data Availability

The raw data supporting the conclusions of this article will be made available by the authors, without undue reservation.

## References

[B1] Alexander S. P. Kelly E. Marrion N. V. Peters J. A. Faccenda E. Harding S. D. (2017). The concise guide to pharmacology 2017/18: Overview. Br. J Pharmacol. 174, S1–S16. 10.1111/bph.13882 29055037 PMC5650665

[B2] Bagur R. Hajnóczky G. (2017). Intracellular Ca^2+^ sensing: Its role in calcium homeostasis and signaling. Mol. Cell 66 (6), 780–788. 10.1016/j.molcel.2017.05.028 28622523 PMC5657234

[B3] Bian J. S. Yong Q. C. Pan T. T. Feng Z. N. Ali M. Y. Zhou S. (2006). Role of hydrogen sulfide in the cardioprotection caused by ischemic preconditioning in the rat heart and cardiac myocytes. J. Pharmacol. Exp. Ther. 316 (2), 670–678. 10.1124/jpet.105.092023 16204473

[B4] Bradley J. M. Organ C. L. Lefer D. J. (2016). Garlic-derived organic polysulfides and myocardial protection. J. Nutr. 146 (2), 403S–409S. 10.3945/jn.114.208066 26764335 PMC4725427

[B5] Braunwald E. Kloner R. A. (1985). Myocardial reperfusion: a double-edged sword? J. Clin. Invest. 76 (5), 1713–1719. 10.1172/JCI112160 4056048 PMC424191

[B6] Bulluck H. Yellon D. M. Hausenloy D. J. (2016). Reducing myocardial infarct size: challenges and future opportunities. Heart 102 (5), 341–348. 10.1136/heartjnl-2015-307855 26674987 PMC4789695

[B7] Chaffman M. Brogden R. N. (1985). Diltiazem: A review of its pharmacological properties and therapeutic efficacy. Drugs 29 (5), 387–454. 10.2165/00003495-198529050-00001 3891302

[B8] Chan J. Y. Yuen A. C. Chan R. Y. Chan S. (2013). A review of the cardiovascular benefits and antioxidant properties of allicin. Phytotherapy Res. 27 (5), 637–646. 10.1002/ptr.4796 22888009

[B9] Chang L. Wang Z. Ma F. Tran B. Zhong R. Xiong Y. (2019). ZYZ-803 mitigates endoplasmic reticulum Stress-related necroptosis after acute myocardial infarction through downregulating the RIP3-CaMKII signaling pathway. Oxidative Med. Cell. Longev. 2019, 6173685. 10.1155/2019/6173685 PMC6589311 31281585

[B10] Chuah S. C. Moore P. K. Zhu Y. Z. (2007). *S* -allylcysteine mediates cardioprotection in an acute myocardial infarction rat model via a hydrogen sulfide-mediated pathway. Am. J. Physiology-Heart Circulatory Physiology 293 (5), H2693–H2701. 10.1152/ajpheart.00853.2007 17766469

[B11] Cui T. Liu W. Chen S. Yu C. Li Y. Zhang J.-Y. (2020). Antihypertensive effects of allicin on spontaneously hypertensive rats via vasorelaxation and hydrogen sulfide mechanisms. Biomed. & Pharmacother. 128, 110240. 10.1016/j.biopha.2020.110240 32480217

[B12] Cui T. Liu W. Yu C. Ren J. Li Y. Shi X. (2022). Protective effects of allicin on acute myocardial infarction in rats via hydrogen sulfide-mediated regulation of coronary arterial vasomotor function and myocardial calcium transport. Front. Pharmacol. 12, 752244. 10.3389/fphar.2021.752244 35046802 PMC8762278

[B14] Donnarumma E. Trivedi R. K. Lefer D. J. (2017). Protective actions of H_2_S in acute myocardial infarction and heart failure. Compr. Physiol. 7 (2), 583–602. 10.1002/cphy.c160023 28333381

[B15] El-Sayed S. S. Shahin R. M. Fahmy A. Elshazly S. M. (2021). Quercetin ameliorated remote myocardial injury induced by renal ischemia/reperfusion in rats: Role of Rho-kinase and hydrogen sulfide. Life Sci. 287, 120144. 10.1016/j.lfs.2021.120144 34785193

[B16] Enokido Y. Suzuki E. Iwasawa K. Namekata K. Okazawa H. Kimura H. (2005). Cystathionine beta-synthase, a key enzyme for homocysteine metabolism, is preferentially expressed in the radial glia/astrocyte lineage of developing mouse CNS. FASEB J. 19 (13), 1854–1856. 10.1096/fj.05-3724fje 16160063

[B17] Fattahi M. Dalir-Naghadeh B. Maham M. (2013). Prophylactic and therapeutic effects of garlic extract on *Nerium oleander* -induced arrhythmia: a new approach to antiarrhythmic therapy in an ovine model. Clin. Toxicol. 51 (8), 737–747. 10.3109/15563650.2013.829234 23944745

[B18] Frangogiannis N. G. (2015). Pathophysiology of myocardial infarction. Compr. Physiol. 5, 1841–1875. 10.1002/cphy.c150006 26426469

[B19] Gao T. Yang P. Fu D. Liu M. Deng X. Shao M. (2021). The protective effect of allicin on myocardial ischemia-reperfusion by inhibition of Ca^2+^ overload-induced cardiomyocyte apoptosis via the PI3K/GRK2/PLC-γ/IP3R signaling pathway. Aging 13 (15), 19643–19656. 10.18632/aging.203375 34343971 PMC8386544

[B20] Han J. Tan H. Duan Y. Chen Y. Zhu Y. Zhao B. (2019). The cardioprotective properties and the involved mechanisms of NaoXinTong Capsule. Pharmacol. Res. 141, 409–417. 10.1016/j.phrs.2019.01.024 30660824

[B21] Hu H. Jiang M. Cao Y. Zhang Z. Jiang B. Tian F. (2020). HuR regulates phospholamban expression in isoproterenol-induced cardiac remodelling. Cardiovasc. Res. 116 (5), 944–955. 10.1093/cvr/cvz205 31373621 PMC7868665

[B22] Khatua T. N. Adela R. Banerjee S. K. (2013). Garlic and cardioprotection: insights into the molecular mechanisms. Can. J. Physiol. Pharmacol. 91 (6), 448–458. 10.1139/cjpp-2012-0315 23746107

[B23] Kimura H. (2017). Hydrogen sulfide and polysulfide signaling. Antioxid. Redox Signal 27 (10), 619–621. 10.1089/ars.2017.7076 28558483

[B24] Laver D. R. (2007). Ca^2+^ stores regulate ryanodine receptor Ca^2+^ release channels via luminal and cytosolic Ca^2+^ sites. Biophysical J. 92 (10), 3541–3555. 10.1529/biophysj.106.099028 PMC1853142 17351009

[B25] Lawson L. Hunsaker S. (2018). Allicin bioavailability and bioequivalence from garlic supplements and garlic foods. Nutrients 10, 812. 10.3390/nu10070812 29937536 PMC6073756

[B26] Lei Y. Liu C. Sheen L. Chen H. Lii C. (2010). Diallyl disulfide and diallyl trisulfide protect endothelial nitric oxide synthase against damage by oxidized low‐density lipoprotein. Mol. Nutr. Food Res. 54, S42–S52. 10.1002/mnfr.200900278 20229525

[B27] Li J. Teng X. Jin S. Dong J. Guo Q. Tian D. (2019). Hydrogen sulfide improves endothelial dysfunction by inhibiting the vicious cycle of NLRP3 inflammasome and oxidative stress in spontaneously hypertensive rats. J. Hypertens. 37 (8), 1633–1643. 10.1097/HJH.0000000000002101 31058793

[B28] Li N. Wang M. J. Jin S. Bai Y. D. Hou C. L. Ma F. F. (2016). The H _2_ S donor NaHS changes the expression pattern of H _2_ S-producing enzymes after myocardial infarction. Oxidative Med. Cell. Longev. 2016, 1–11. 10.1155/2016/6492469 PMC4736414 27057284

[B29] Ma L. Li L. Li S. Hao X. Zhang J. He P. (2017). Allicin improves cardiac function by protecting against apoptosis in rat model of myocardial infarction. Chin. J. Integr. Med. 23 (8), 589–597. 10.1007/s11655-016-2523-0 27412589

[B30] MacLeod K. T. (2016). Recent advances in understanding cardiac contractility in health and disease. F1000Res 5, F1000 Faculty Rev-1770. 10.12688/f1000research.8661.1

[B31] Matsumoto T. Miura T. Miki T. Nishino Y. Nakamura Y. Shimamoto K. (2003). Does enhanced expression of the Na^+^-Ca^2+^ exchanger increase myocardial vulnerability to ischemia/reperfusion injury in rabbit hearts? Mol. Cell Biochem. 248 (1-2), 141–147. 10.1023/a:1024140419688 12870666

[B32] Mattiazzi A. Kranias E. G. (2014). The role of CaMKII regulation of phospholamban activity in heart disease. Front. Pharmacol. 5, 5. 10.3389/fphar.2014.00005 24550830 PMC3913884

[B33] McAloon C. J. Boylan L. M. Hamborg T. Stallard N. Osman F. Lim P. B. (2016). The changing face of cardiovascular disease 2000–2012: An analysis of the world health organisation global health estimates data. Int. J. Cardiol. 224, 256–264. 10.1016/j.ijcard.2016.09.026 27664572

[B34] Mocayar M. F. J. Camargo A. B. Manucha W. (2020). Allicin pharmacology: Common molecular mechanisms against neuroinflammation and cardiovascular diseases. Life Sci. 249, 117513. 10.1016/j.lfs.2020.117513 32145307

[B35] Pearle D. L. (1988). Calcium antagonists in acute myocardial infarction. Am. J. Cardiol. 61 (3), 22–25. 10.1016/0002-9149(88)91351-3 3277363

[B36] Peng S. Zhao D. Li Q. Wang M. Zhang S. Pang K. (2022). Hydrogen sulfide regulates SERCA2a ubiquitylation via muscle RING finger-1 S-sulfhydration to affect cardiac contractility in db/db mice. Cells 11 (21), 3465. 10.3390/cells11213465 36359861 PMC9658184

[B37] Petrosino M. Zuhra K. Kopec J. Hutchin A. Szabo C. Majtan T. (2022). H_2_S biogenesis by cystathionine beta-synthase: mechanism of inhibition by aminooxyacetic acid and unexpected role of serine. Cell Mol. Life Sci. 79 (8), 438. 10.1007/s00018-022-04479-9 35864237 PMC9304066

[B38] Pott C. Eckardt L. Goldhaber J. I. (2011). Triple threat: the Na^+^/Ca^2+^ exchanger in the pathophysiology of cardiac arrhythmia, ischemia and heart failure. Curr. Drug Targets 12 (5), 737–747. 10.2174/138945011795378559 21291388 PMC4406235

[B39] Reyes Gaido O. E. Nkashama L. J. Schole K. L. Wang Q. Umapathi P. Mesubi O. O. (2023). CaMKII as a therapeutic target in cardiovascular disease. Annu. Rev. Pharmacol. Toxicol. 63 (1), 249–272. 10.1146/annurev-pharmtox-051421-111814 35973713 PMC11019858

[B40] Rose C. R. Ziemens D. Verkhratsky A. (2020). On the special role of NCX in astrocytes: Translating Na^+^-transients into intracellular Ca^2+^ signals. Cell Calcium 86, 102154. 10.1016/j.ceca.2019.102154 31901681

[B41] Song Z. Song H. Liu D. Yan B. Wang D. Zhang Y. (2022). Overexpression of MFN2 alleviates sorafenib-induced cardiomyocyte necroptosis via the MAM-CaMKIIδ pathway *in vitro* and *in vivo* . Theranostics 12 (3), 1267–1285. 10.7150/thno.65716 35154486 PMC8771548

[B42] Sungnoon R. Kanlop N. Chattipakorn S. C. Tawan R. Chattipakorn N. (2008). Effects of garlic on the induction of ventricular fibrillation. Nutrition 24 (7-8), 711–716. 10.1016/j.nut.2008.03.003 18456459

[B43] Talaei F. Bouma H. R. Van Der Graaf A. C. Strijkstra A. M. Schmidt M. Henning R. H. (2011). Serotonin and dopamine protect from hypothermia/rewarming damage through the CBS/H2S pathway. PLoS ONE 6 (7), e22568. 10.1371/journal.pone.0022568 21829469 PMC3144905

[B44] Tomita M. Nagahara N. Ito T. (2016). Expression of 3-mercaptopyruvate sulfurtransferase in the mouse. Molecules 21 (12), 1707. 10.3390/molecules21121707 27973427 PMC6273466

[B45] Valentim M. A. Brahmbhatt A. N. Tupling A. R. (2022). Skeletal and cardiac muscle calcium transport regulation in health and disease. Biosci. Rep. 42 (12), BSR20211997. 10.1042/BSR20211997 36413081 PMC9744722

[B46] Wang R. (2012). Physiological implications of hydrogen sulfide: a whiff exploration that blossomed. Physiol. Rev. 92 (2), 791–896. 10.1152/physrev.00017.2011 22535897

[B47] Wayman G. A. Tokumitsu H. Davare M. A. Soderling T. R. (2011). Analysis of CaM-kinase signaling in cells. Cell Calcium 50 (1), 1–8. 10.1016/j.ceca.2011.02.007 21529938 PMC3236032

[B48] Wei X. Jin J. Wu J. He Y. Guo J. Yang Z. (2023). Cardiac-specific BACH1 ablation attenuates pathological cardiac hypertrophy by inhibiting the Ang II type 1 receptor expression and the Ca^2+^/CaMKII pathway. Cardiovasc. Res. 119 (9), 1842–1855. 10.1093/cvr/cvad086 37279500

[B49] Wen Y. D. Wang H. Zhu Y. Z. (2018). The drug developments of hydrogen sulfide on cardiovascular disease. Oxidative Med. Cell. Longev. 2018, 4010395. 10.1155/2018/4010395 PMC6087600 30151069

[B50] Wollert K. C. Drexler H. (2010). Cell therapy for the treatment of coronary heart disease: a critical appraisal. Nat. Rev. Cardiol. 7 (4), 204–215. 10.1038/nrcardio.2010.1 20177405

[B51] Wu D. Hu Q. Tan B. Rose P. Zhu D. Zhu Y. Z. (2018). Amelioration of mitochondrial dysfunction in heart failure through S-sulfhydration of Ca^2+^/calmodulin-dependent protein kinase II. Redox Biol. 19, 250–262. 10.1016/j.redox.2018.08.008 30195191 PMC6128039

[B52] Xu W. Li X. Li E. Liu Y. Zhao J. Wei L. (2020). Protective effects of allicin on ISO-induced rat model of myocardial infarction via JNK signaling pathway. Pharmacology 105 (9-10), 505–513. 10.1159/000503755 32784309

[B53] Xue J. Zeng W. Han Y. John S. Ottolia M. Jiang Y. (2023). Structural mechanisms of the human cardiac sodium-calcium exchanger NCX1. Nat. Commun. 14 (1), 6181. 10.1038/s41467-023-41885-4 37794011 PMC10550945

[B54] Yang G. Sun X. Wang R. (2004). Hydrogen sulfide-induced apoptosis of human aorta smooth muscle cells via the activation of mitogen-activated protein kinases and caspase-3. FASEB J. 18 (14), 1782–1784. 10.1096/fj.04-2279fje 15371330

[B55] Yellon D. M. Hausenloy D. J. (2007). Myocardial reperfusion injury. N. Engl. J. Med. 357 (11), 1121–1135. 10.1056/NEJMra071667 17855673

[B56] Zhang H. Bai Z. Zhu L. Liang Y. Fan X. Li J. (2020). Hydrogen sulfide donors: therapeutic potential in anti-atherosclerosis. Eur. J. Med. Chem. 205, 112665. 10.1016/j.ejmech.2020.112665 32795766

[B57] Zhang Y. Jiao L. Sun L. Li Y. Gao Y. Xu C. (2018). LncRNA *ZFAS1* as a SERCA2a inhibitor to cause intracellular Ca^2+^ overload and contractile dysfunction in a mouse model of myocardial infarction. Circulation Res. 122 (10), 1354–1368. 10.1161/CIRCRESAHA.117.312117 29475982 PMC5959220

[B58] Zhao P. Zhou W. Zhang Y. Li J. Zhao Y. Pan L. (2020). Aminooxyacetic acid attenuates post-infarct cardiac dysfunction by balancing macrophage polarization through modulating macrophage metabolism in mice. J. Cell Mol. Med. 24 (4), 2593–2609. 10.1111/jcmm.14972 31930778 PMC7028849

[B59] Zhao W. Zhang J. Lu Y. Wang R. (2001). The vasorelaxant effect of H(2)S as a novel endogenous gaseous K(ATP) channel opener. EMBO J. 20 (21), 6008–6016. 10.1093/emboj/20.21.6008 11689441 PMC125693

[B60] Zhao Y. Gao F. Zhang Y. Wang H. Zhu J. Chang L. (2016). Shensong Yangxin capsules prevent ischemic arrhythmias by prolonging action potentials and alleviating Ca^2+^ overload. Mol. Med. Rep. 13 (6), 5185–5192. 10.3892/mmr.2016.5203 27122298

[B61] Zhihao L. Jingyu N. Lan L. Michael S. Rui G. Xiyun B. (2020). SERCA2a: a key protein in the Ca^2+^ cycle of the heart failure. Heart Fail Rev. 25 (3), 523–535. 10.1007/s10741-019-09873-3 31701344

[B62] Zhu Y. Anand R. Geng X. Ding Y. (2018). A mini review: garlic extract and vascular diseases. Neurological Res. 40 (6), 421–425. 10.1080/01616412.2018.1451269 29557277

[B63] Zuhra K. Augsburger F. Majtan T. Szabo C. (2020). Cystathionine-β-synthase: molecular regulation and pharmacological inhibition. Biomolecules 10 (5), 697. 10.3390/biom10050697 32365821 PMC7277093

