# Supplementary material for: Allicin ameliorates acute myocardial infarction in rats by modulating calcium homeostasis in cardiomyocytes through the induction of hydrogen sulfide production
Source: Front Pharmacol. 2025 Mar 26;16:1557685. doi: 10.3389/fphar.2025.1557685 (PMC11979285; doi:10.3389/fphar.2025.1557685)
Supplement: Supplementary file 1 [file DataSheet1.docx]

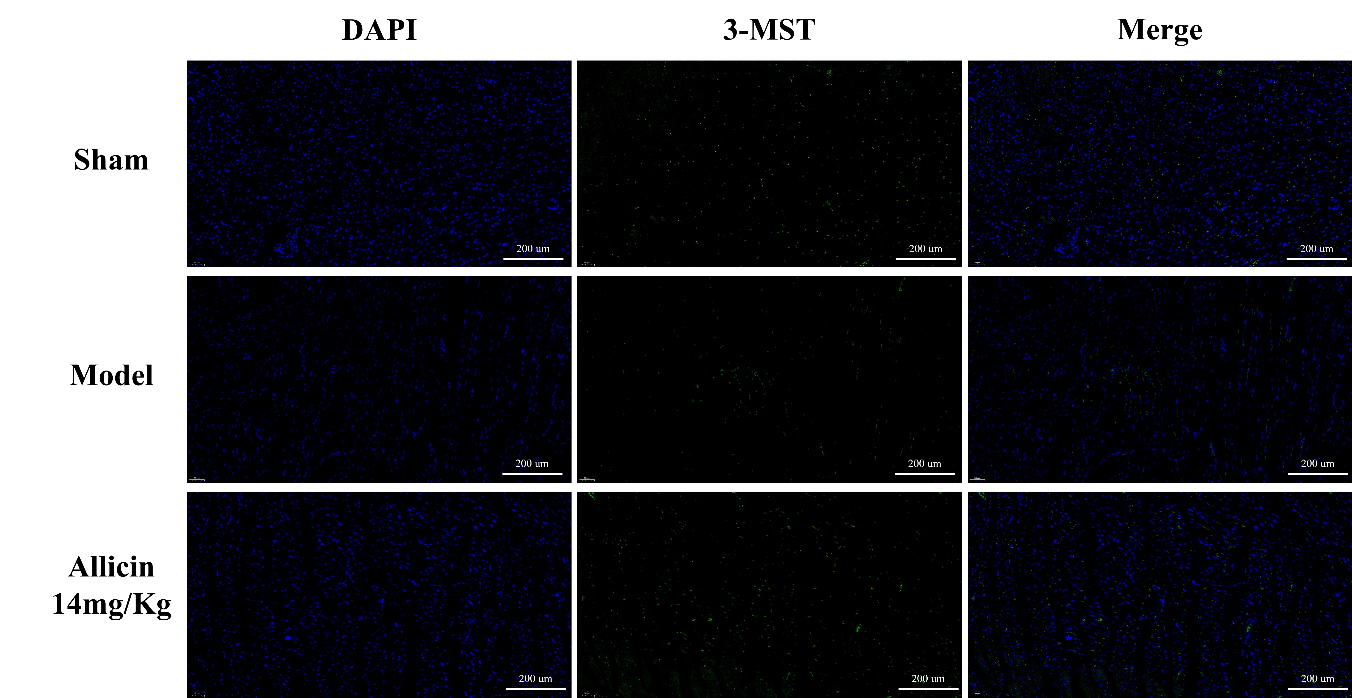


**Supplementary Figure 1. Fluorescence images of representative 3-MST of each group in myocardial tissue, Scale bar = 200 um (n = 3).**
